# Impact of soft tissue augmentation procedures on esthetics and patient satisfaction in the treatment of peri‐implant buccal soft tissue dehiscences: A systematic review and meta‐analysis

**DOI:** 10.1111/prd.12633

**Published:** 2025-07-17

**Authors:** José Nart, Cristina Valles, Javi Vilarrasa, Federica Romano, Giacomo Baima, Mario Aimetti

**Affiliations:** ^1^ Department of Periodontology Universitat Internacional de Catalunya Barcelona Spain; ^2^ Department of Surgical Sciences University of Turin Turin Italy

**Keywords:** connective tissue graft, coronally advanced flap, dental implants, esthetics, meta‐analysis, soft tissue implant interaction

## Abstract

The aim of this systematic review was to assess the performance of soft tissue augmentation (STA) procedures, with or without a modification of the prosthetic rehabilitation, for the treatment of buccal peri‐implant soft tissue dehiscence (PSTD) in terms of esthetics and patient‐reported outcomes. A systematic review protocol was developed following the PRISMA checklist. Electronic and hand searches were conducted to identify randomized clinical trials (RCTs) and prospective studies on the treatment of buccal PSTD in implants without peri‐implantitis, with a follow‐up of at least 6 months. Professional assessment of esthetics and self‐reported patient satisfaction were considered the primary outcomes, while clinical variables were considered secondary outcomes. Meta‐analysis was carried out when possible using a fixed‐ or random‐effect model. Eight publications reporting on five studies (two RCTs and three prospective studies), published from 2013 to 2024 and including a total of 87 patients, were included in this systematic review. All studies evaluated a coronally advanced flap (CAF) with connective tissue graft (CTG) or substitutes, whereas one arm of an RCT employed a tunnel procedure. Two studies included changing of the prosthetic component. Three studies were rated at low risk of bias. A total of 10 meta‐analyses were performed. STA using CAF achieved a final professional esthetic score of 7.7 on a 0–10 scale (95% CI: 6.63; 8.83) and showed improvements in terms of patient‐reported esthetics on a 0–100 visual analogue scale (60.8; 95% CI: 46.56; 75.01), with moderate‐to‐high heterogeneity. The estimated reduction in PSTD depth was 2.2 mm (95% CI: 1.76; 2.69), with an estimated rate of complete PSTD coverage of 71% (95% CI: 59; 82). Based on limited evidence, it can be concluded that STA procedures around implants affected by buccal PSTD appear to positively influence both professional and patient‐reported esthetics outcomes.

## 1 INTRODUCTION

Peri‐implant soft tissue dehiscence (PSTD), also referred as mid‐facial recession, mucosal recession, or peri‐implant soft tissue recession, can be defined as the apical shift of the mucosal margin exposing part of the prosthetic or implant components.^1^ The prevalence of PSTDs was estimated around 16.9%, being 12% when only implants without peri‐implantitis are considered.^2^ The display of the implant component(s) through the mucosa or a discrepancy in the length of implant crown have been also referred as PSTDs.^3^ This condition, which is commonly observed on the facial aspect, may not only compromise peri‐implant health in the long term,^4^ but it can also impact the esthetic appearance of the peri‐implant tissues/restorations and the overall patient satisfaction.^5^

Several surgical (i.e., bucco‐oral malposition, depth of the implant platform), site‐specific (i.e., buccal bone‐level thickness, peri‐implant phenotype, width and thickness of keratinized mucosa) and prosthetic (i.e., type of restorations, prosthetic design) factors have been associated with the occurrence of PSTD in implants not suffering peri‐implantitis.^2^, ^6^, ^7^, ^8^ In fact, a recent meta‐analysis evidenced that buccally placed implants and thin phenotypes were the factors most associated with PSTD in the absence of peri‐implantitis, increasing the risk by 14.37‐ and 2.85‐fold, respectively.^1^

When the implant affected is not deemed for explantation, the management of PSDT may involve the combination of soft tissue augmentation procedures*—*using autogenous (i.e., epithelized free gingival graft or connective tissue graft) or soft tissue substitutes (i.e., acellular dermal matrix or xenogeneic collagen matrix) with different flap techniques [i.e., coronally advanced flap via envelope, tunnel or VISTA (Vestibular Incision Subperiosteal Tunnel Access)]*—*together with prosthetic modifications, if needed.^9^

In recent studies, soft tissue reconstructive interventions for treating PSTD achieved favorable clinical outcomes in terms of complete coverage,^10^ leading to a pleasant esthetic appearance^11^ and high levels of patient satisfaction.^11^, ^12^ However, the performance of soft tissue augmentation for the treatment of PSTD in terms of esthetics and patient‐reported outcomes needs to be systematically analyzed.

Therefore, the primary objective of this systematic review is to address the following focused question, formulated using the PIOS (Patient, Intervention, Outcome and Study Design) format: In systemically healthy humans with at least one dental implant with a PSTD in absence of peri‐implantitis (population), what is the performance of soft tissue augmentation procedures with or without a modification of the prosthetic rehabilitation (intervention), in terms of professionally assessment of esthetics and self‐reported patient satisfaction (primary outcome), as reported in randomized controlled clinical trials, controlled clinical trials, prospective studies—cohort studies and case series—with a minimum of 10 patients and at least 6 months of follow‐up (study design)?

## 2 MATERIALS AND METHODS

This study was conducted and reported following the PRISMA (Preferred Reporting Items for Systematic Reviews and Meta‐analyses) statement.^13^ The protocol was originally registered in the International Prospective Register of Systematic Reviews (PROSPERO) (CRD42024547627).

### 2.1 Eligibility criteria

#### 2.1.1 Inclusion criteria for studies

- *Population*. Systemically healthy humans, older than 18 years, with at least one dental implant loaded with fixed or removable prostheses, requiring soft tissue reconstruction due to a PSTD in the absence of peri‐implantitis.
- *Intervention*. Any type of soft tissue augmentation procedure aimed at coverage of PSTD, with or without a modification of the prosthetic rehabilitation.
- *Outcome*. Studies reporting data on at least the primary outcome variable: professional assessment of esthetics and patient‐related outcomes (PROMs) (i.e., esthetics and patient satisfaction). Moreover, PROMs in terms of morbidity, postoperative complications, surgical time, and costs were considered secondary outcomes. Other secondary outcomes were the following: changes in the position of the peri‐implant soft tissues (level of the mucosal margin and height of the papilla), mean and complete coverage of buccal soft tissue dehiscence, changes in the dimension of keratinized mucosa, changes in the peri‐implant soft tissue color, changes in the peri‐implant tissue health status [probing depth (PD), clinical attachment level (CAL), plaque indices, bleeding indices], marginal bone‐level changes, volumetric changes of the peri‐implant soft tissue, and changes in mucosal phenotype.
- *Study design*. Randomized controlled clinical trials (RCTs) and controlled clinical trials (CCTs) with a minimum of 10 patients (5 patients per group) as well as prospective studies (i.e., cohort studies or case series) and retrospective studies with a minimum of 10 patients and at least 6 months of follow‐up after the surgical intervention.

#### 2.1.2 Exclusion criteria for studies

- Studies that combined guided bone regeneration and soft tissue augmentation procedures;
- Studies evaluating coverage of buccal soft tissue recessions at teeth;
- Soft tissue augmentation performed at implant placement;
- Implants affected by peri‐implantitis;
- Single case reports, animal experiments, and ex vivo or in vitro studies;
- Studies published in languages other than English were excluded due to time constraints.

### 2.2 Search strategy

An electronic search was performed in the following online databases for studies published until April 2024: (i) The National Library of Medicine (MEDLINE via PubMed); (ii) Scopus; (iii) Cochrane Central Register of Controlled Trials; and (iv) Embase.

The search strategy included the use of MeSH terms as well as text words and Boolean operators (OR, AND) to combine searches. Detailed search strategies were created for each database searched (Appendix 1). Additionally, the reference lists of selected studies and previously published systematic reviews were manually examined to identify further relevant papers. Hand searching was performed for the following journals: *Journal of Clinical Periodontology, Journal of Periodontology, Journal of Periodontal Research, Clinical Implant Dentistry and Related Research*, *and The International Journal of Oral & Maxillofacial Implants*.

### 2.3 Study selection

Before starting the screening process, two reviewers (FR and GB) underwent calibration. A meeting was held to discuss the eligibility criteria, followed by screening a random sample of 50 citations (i.e., 5 blocks of 10 studies) based on title and abstract. Any discrepancies were resolved through discussion with the other reviewers. Both reviewers (FR and GB) were calibrated once full agreement was achieved. The same criteria were applied during the full‐text calibration. All search results were managed using Rayyan software,^14^ and duplicate manuscripts were subsequently removed.

In the first stage, two reviewers (FR and GB) independently screened the studies by reviewing the titles and abstracts from both electronic and manual searches. The main reasons for excluding studies at this or any subsequent stage were recorded using a PIO elements coding scheme developed before the screening process. If the title and abstract did not provide sufficient information to determine inclusion or exclusion, the full text was obtained along with the selected articles. Studies selected by at least one reviewer were included in the full‐text analysis. In the second stage, the selected studies were independently full‐text reviewed by both reviewers (FR and GB) based on the inclusion and exclusion criteria. Any disagreements were resolved through consultation with the other reviewers.

Moreover, in cases of overlapping data or duplicate publications, the most comprehensive data were preferentially included. Although no language restrictions were imposed, only studies published in English were considered due to time constraints.

The inter‐reviewer reliability, including the percentage of agreement and the kappa coefficient, was calculated at both stages of the screening process.

### 2.4 Data extraction

Two reviewers (CV and JV) independently extracted the data from the included studies and compiled it into a standardized data extraction form, which was developed and reviewed by all authors. Any discrepancies in the extracted data were resolved through discussion between the reviewers. In cases of overlapping data between different publications on the same study, the most inclusive data were preferentially selected for data extraction.

To obtain additional or missing information, the corresponding authors were contacted twice by email within a 1‐week period. Data were excluded until clarification was provided.

Data extracted focused on general study characteristics (authors and year of publication, study design, follow‐up period, setting, unit of analysis, augmentation procedures, and funding), population characteristics (age, gender, smoking status, and number of baseline and final patients), and implant/surgical features (brand, diameter, location, donor site, PSTD type, restoration type, and prosthetic therapy). Additionally, data on the primary and secondary outcomes were extracted.

### 2.5 Quality assessment of the included studies

Quality assessment was independently conducted by two reviewers (CV and GB). The risk of bias in randomized clinical trials was evaluated using the RoB 2 tool.^15^ The following domains were assessed: sequence generation, allocation concealment, blinding of participants, personnel and outcome assessors, incomplete outcome data, and selective outcome reporting. Additional potential risks, such as the funding source, were also considered. The risk of bias for each domain was categorized as lower, unclear, or high.

The risk of bias of prospective case series was assessed by the National Institutes of Health's Quality Assessment Tool for Case Series Studies.^16^ This tool presents nine questions with an overall rating of good, fair, or poor.

Any disagreements were resolved through discussion between the two reviewers. Inter‐reviewer reliability in the quality assessment was evaluated by calculating the percentage of agreement and the kappa coefficient.

### 2.6 Data analysis

For both primary and secondary outcomes, data reporting mean values and their standard deviations (SD) [or standard errors of the mean (SEM) if SD was unavailable] were extracted from each study. An overall effect size and 95% confidence intervals (CIs) were subsequently calculated. For studies using visual analogue scales (VAS) of different magnitudes, values were converted to a standardized VAS of 0–100. Study‐specific estimates were pooled using fixed‐ or random‐effect models, and forest plots were generated to visually represent the meta‐analysis.

The Cochran‐Q test^17^ was performed to assess heterogeneity among studies, and the *I* ^2^ index^18^ was used to describe the percentage of variation across studies attributed to heterogeneity (*I* ^2^ = 25%: low; *I* ^2^ = 50%: moderate; *I* ^2^ = 75%: high).

All statistical analyses were conducted using OpenMeta[Analist] (Brown University, RI, USA) software. Statistical significance was set as *p* value <0.05.

## 3 RESULTS

### 3.1 Study selection

**FIGURE 1:**
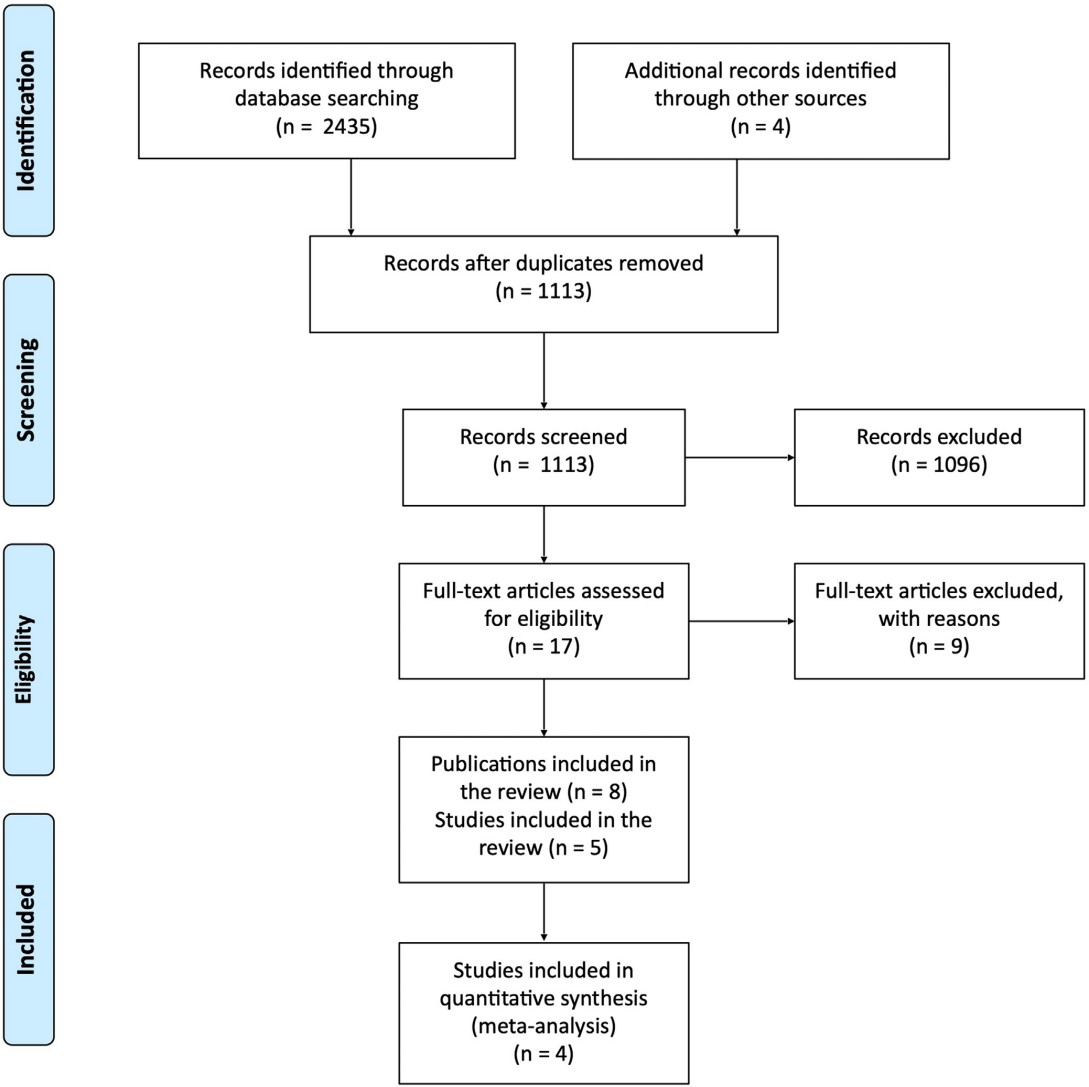
PRISMA flow chart illustrating the search strategy that led to the inclusion of eight publications corresponding to five studies.

The study selection process is depicted in the PRISMA flow diagram (Figure 1), with detailed reasons for exclusion provided in Appendix 2. Initially, the search of electronic databases identified 2435 records, with an additional 4 records found through other sources. After removing duplicates, 1113 studies were screened by title and abstract, of which 1096 were excluded. Accordingly, 17 full‐text articles were obtained. Following a thorough review, 9 studies were excluded for not meeting the inclusion criteria. Ultimately, 8 articles were included for data extraction, representing 5 studies [2 publications^12^, ^19^ and 3 publications^20^, ^21^, ^22^ showed different follow‐ups from the same population]. Inter‐examiner reliability was excellent in both the screening and inclusion process (*k* score = 0.87, 95% CI: [0.83–0.91]) and the full‐text appraisal (*k* score = 0.92, 95% CI: [0.89–0.95]).

### 3.2 Study characteristics

#### 3.2.1 Study design and groups

Table 1 describes an overview of the relevant characteristics of the selected studies. All studies were published between 2013 and 2024. Two studies were two‐arm RCT,^23^, ^24^ one was a prospective cohort study,^19^ one a prospective case series^25^ and another was described as a prospective study.^22^ Three studies were conducted in a university setting,^19^, ^24^, ^25^ one in a private practice^22^ and one involved both a university and a private practice setting.^23^ The interventions performed were as follows: coronally advanced flap (CAF) + connective tissue graft (CTG),^19^, ^23^, ^24^ CAF + acellular dermal matrix (ADM),^23^ partial thickness CAF without releasing vertical incisions + CTG,^22^ CAF with two vertical releasing incisions + CTG^25^ and TUN + CTG.^24^ It should be noted that since the study by Tavelli et al.^24^ included two groups (i.e., CAF and TUN), only the CAF group was considered for inclusion in the meta‐analyses to avoid introducing heterogeneity into the results.

**TABLE 1:** Methodological characteristics of the selected studies.

References	Year of publication	Study design	Follow‐up (months)	Setting	Level of analysis	Test group	Control group	Funding
Anderson et al.	2014	RCT	6	University and private practice	Patient	CAF + ADM	CAF + CTG	Research Fund (University of Michigan). Material donated by BioHorizons®
Zucchelli et al.	2013, 2018	Prospective cohort	60	University	Patient	CAF + CTG	—	NR
Roccuzzo et al.	2014, 2019, 2024	Prospective	120	Private practice	Patient	Partial‐thickness CAF without vertical incisions + CTG	—	Self‐supported
Tavelli et al. (a)	2023	RCT	12	University	Patient	CAF + CTG	TUN + CTG	Grant from the Delta Dental Foundation
Tavelli et al. (b)	2023	Prospective case series	12	University (unclear)	Patient	CAF with two vertical incisions +2 CTGs for horizontal and vertical augmentation	—	Self‐supported

Abbreviation: ADM, acellular dermal matrix allograft; CAF, coronally advanced flap technique; CTG, connective tissue graft; NR, not reported; RCT, randomized clinical trial; TUN, tunnel technique.

#### 3.2.2 Study population and follow‐up

Details regarding patient characteristics are presented in Table 2. The included studies provided data for 87 patients. Sample sizes varied from 10 patients^25^ to 28 subjects.^24^ The duration of the follow‐up ranged from 6 months^23^ to 120 months^21^ (Table 1).

**TABLE 2:** Sample characteristics of included studies.

References	Mean age (range) Test/Control *Global (years)	Gender (% female) Test/Control *Global	Smoking habit (%) Test/Control *Global	Baseline patients (PSTD) Test/Control	Final patients (PSTD) Test/Control
Anderson et al.	NR	NR	Inclusion criteria: Smoking ≤10 cig/day. *N* smokers was not reported	13 (13) 6/7	13 (13) 6/7
Zucchelli et al.	(26–53)	70%	Inclusion criteria: Smoking <10 cig/day. *N* smokers was not reported	20 (20)	19 (19)
Roccuzzo et al.	53.1 ± 11.7	81.25%	18.8%. Heavy smokers (> 15 cig/day) were excluded	16 (16)	12 (12)
Tavelli et al. (a)	CAF: 46.9 ± 9.8 TUN: 47.1 ± 13.6 *47.0 ± 12.1	CAF: 57.1% TUN: 57.1% *57.1%	Inclusion criteria: Smoking ≤10 cig/day. N former smokers: 1 in CAF/1 in TUN	28 (28) 14/14	28 (28) 14/14
Tavelli et al. (b)	52.8 ± 13.9	70%	Smokers were excluded	10 (10)	10 (10)

*Note*: The data related to age, gender, and smoking habit in the studies by Zucchelli et al.^12^ and Roccuzzo et al.^21^ refer to baseline values.

Abbreviations: CAF, coronally advanced flap technique; NR, not reported; PSTD, peri‐implant soft tissue dehiscence; TUN, tunnel technique.

#### 3.2.3 Implant and intervention characteristics

Table 3 outlines the implant and intervention characteristics of selected studies.

**TABLE 3:** Implant and intervention characteristics of selected studies.

References	Implant brand	Implant diameter (mm)	Implant site location	Soft tissue grafting (functional loading, years)	Donor site	PSTD type^a^	Restoration type	Prosthetic therapy
Anderson et al.	NR	NR	Maxilla, non‐molar	NR	NR	NR	NR	NR
Zucchelli et al.	NR	NR	Maxilla, anterior (14 to 24)	PSTD at the buccal aspect after more than two years of loading	Lateral palate with extraoral de‐epithelization. Thickness of the graft: ≈ 2 mm	NR	NR (provisional restoration was cemented)	The implant crown was removed, the abutment reduced and polished, and a short provisional crown placed. Final restorations were performed eight months after the surgical intervention.
Roccuzzo et al.	Straumann® TL (collar length: 1.8, 2.8 mm)	4.1, 3.3	Maxilla, anterior and posterior	PSTD in functionally loaded dental implants (i.e., loaded from 1.2 to 10 years)	Tuberosity with extraoral de‐epithelization	NR	Cemented	NR
Tavelli et al. (a)	NR (bone‐level implants)	NR	Maxilla and mandible, non‐molar	PSTD in functionally loaded dental implants (i.e., prosthetically loaded for at least one year)	Lateral palate with extraoral de‐epithelization. Thickness of the graft: 1.5–2 mm	13 diagnosed as IIa and 15 as IIb	NR	Non‐removal of the restorations
Tavelli et al. (b)	NR (bone‐level implants)	NR	Maxilla, anterior	8.6 ± 2.7	Lateral palate and tuberosity (if available) with extraoral de‐epithelization). Platelet rich fibrin membranes over the CTG	8 diagnosed as IVc and 2 as IIIc	NR	The implant crown and abutment were removed and replaced with a cover screw. For buccally positioned implants, <1.5 mm apart from the adjacent tooth with interproximal attachment loss and adjacent teeth with crowns or extensive restorations, implants were left submerged and finalize with a fixed prosthesis. Restorable implants were opened after three months, a temporary crown was placed, and final restorations were performed after six months

Abbreviations: CTG, connective tissue graft; NR, not reported; PSTD, peri‐implant soft tissue dehiscence; TL, Tissue Level Implants.

^a^ Classification described by Zucchelli et al.^3^

Implant design and dimensions were only reported in three investigations. Roccuzzo et al.^22^ included one‐piece implants with diameters of 3 mm and 4.1 mm, while Tavelli et al.^24^, ^25^ included two‐piece bone‐level implants.

One study focused on PSTD in the maxillary non‐molar area,^19^, ^23^ one study investigated the maxillary anterior area (i.e., canines and incisors),^25^ another investigation included maxillary anterior and posterior sites^22^ and two studies included maxillary and mandibular non‐molar sites.^24^ The PSTD type was only specified in two studies^24^, ^25^ using the classification previously described by Zucchelli et al.,^3^ including type IIa and IIb^24^ and IVc and IIIc.^25^

Regarding prosthesis characteristics, only one study mentioned that the restoration was cemented.^22^ In addition, implant restorations were not removed in one investigation,^24^ while in the study conducted by Zucchelli et al.^19^ the restoration was removed, the abutment polished, and a short provisional restoration was placed during the healing period. In contrast, Tavelli et al.^25^ removed the restoration and abutment, placed a cover screw for a submerged surgery, and provided provisional restorations after 3 months.

The donor site for CTG harvesting was the lateral palate,^19^, ^24^ the tuberosity^22^ or both tuberosity, if available, and the lateral palate.^25^

### 3.3 Effects of intervention

Tables 4 and 5 display all the relevant outcomes from the included studies.

**TABLE 4:** Patient‐reported outcome measures and professional assessment of aesthetics.

References	PROMs: aesthetics		Professional assessment of aesthetics		PROMs: satisfaction		PROMs: morbidity		Surgical time (min)
	Mean ± SD	Method of assessment	Mean ± SD	Method of assessment	Mean ± SD	Method of assessment	mean ± SD	Method of assessment	mean ± SD
Anderson et al.	CAF + CTG: 3.95 CAF + ADM: 3.39	Survey using the CEI.^26^ Five‐point answer scale	CAF + CTG: 1.20 CAF + ADM: 1.69	CEI^26^		Patients' QoL: revised version of the Kiyak Post‐Surgical Patient Satisfaction Questionnaire^31^	CAF + CTG: 4.43 CAF + AD: 4.67	Survey (5 point‐scale). Morbidity at 14 days post‐operative	NR
Zucchelli et al.	8.95 ± 0.91	VAS (0–10) (Cortellini et al.^27^, ^28^)	PES: 8.84 ± 0.90 PES/WES: 17.63 ± 0.96	PES/WES^29^ evaluated on photographs	NR	NR	NR	NR	NR
Roccuzzo et al.	9.5 ± 0.8	VAS (0–10)	8.5 ± 0.9	VAS (0–10) evaluated on photographs	NR	NR	NR	NR	NR
Tavelli et al. (a)	CAF: 74.4 ± 16 TUN: 43.4 ± 13.5	VAS (0–100)	CAF: 7.29 ± 2.58 TUN: 4.86 ± 2.41	IDES^30^	CAF: 5.83 ± 4.01 TUN: 19.26 ± 6.80	QoL in terms of anxiety	CAF: 18.9 ± 9.5 TUN: 14.2 ± 8.4	VAS (0–100). Post‐operative morbidity during first week after surgery	CAF: 82 ± 8 TUN: 80 ± 5
Tavelli et al. (b)	8.83 ± 1.34	VAS (0–10)	6.9 ± 2.33	IDES^30^	NR	NR	2.63 ± 1.96	VAS (0–10). Post‐operative morbidity during first two weeks after surgery	NR

*Note*: Publications with the longest follow‐up were selected.^12^, ^21^

Abbreviations: ADM, acellular dermal matrix allograft; CAF, coronally advanced flap technique; CEI, Complex Esthetic Index; CTG, connective tissue graft; IDES, Implant soft tissue Dehiscence coverage Esthetic Score; NR, not reported; PES, Pink Esthetic Score; PROMs, patient‐reported outcome measures; QoL, quality of life; SD, standard deviation; TUN, tunnel technique; VAS, Visual Analogue Scale; WES, White Esthetic Score.

**TABLE 5:** Outcomes of the selected studies.

References	PSTD depth (mm)				PSTD coverage (%)	Complete PSTD coverage (%)
	Baseline mean ± SD	Final mean ± SD	Baseline‐Final mean ± SD	Method of assessment	mean ± SD	
Anderson et al.	NR	CAF + CTG: 0.43 CAF + ADM: 0.83	NR	From crown margin	NR	NR
Zucchelli et al.	3 (2–3)^a^ 2.7 ± 0.68	0 (0–0)^a^ 0.03 ± 0.49	3 (2–3)^a^ 2.74 ± 0.81	Obtained by subtracting the clinical crown length of the natural tooth from the distance between the stent reference point and the soft tissue margin. The clinical crown length was determined by measuring from the occlusal margin of the tooth to the gingival margin.	99.2 ± 16.1	79
Roccuzzo et al.	1.9 ± 0.7	0.2 ± 0.2	1.7 ± 0.62	Distance from the implant shoulder to the coronal margin of the mucosa with a Castroviejo Caliper Short.	89.6 ± 17.1	58.3
Tavelli et al. (a)	CAF: 2.46 ± 0.87 TUN: 2.36 ± 0.46	CAF: 0.25 ± 0.47 TUN: 1.00 ± 0.88	CAF: 2.21 ± 0.75 TUN: 1.36 ± 0.76	The corono‐apical distance between the peri‐implant soft tissue margin and the CEJ of the homologous contralateral tooth was measured using customized stents and a periodontal probe^3^, ^19^	CAF: 90.23 ± 19.85 TUN: 59.79 ± 34.94	CAF: 71.4 TUN: 28.6
Tavelli et al. (b)	2.60 ± 0.61	0.35 ± 0.47	2.25 ± 0.82	The corono‐apical distance between the peri‐implant soft issue margin and the ideal position of the soft tissue level (i.e., gingival margin of the contralateral homologous tooth) was measured using a periodontal probe	85.14 ± 21.11	60%

References	Soft tissue thickness (mm)				Volume gain (mm)	Keratinized mucosa width (mm)		
	Baseline mean ± SD	Final mean ± SD	Baseline‐Final mean ± SD	Method of assessment	Baseline‐Final mean ± SD	Baseline mean ± SD	Final mean ± SD	Baseline‐Final mean ± SD
Anderson et al.	NR	NR	CAF + CTG: 1 CAF + ADM: 1.75	Transmucosal thickness of 1 and 3 mm apical to the free gingival margin using bone sounding with endodontic files Triad stents were used for standardization.	NR	NR	NR	NR
Zucchelli et al.	0.9 (0.7–1.1)^a^ 0.93 ± 0.27	2.60 (2.40–3.10)^a^ 2.75 ± 0.40	1.8 (1.60–2.10)^a^ −1.83 ± 0.25	Measured 1.5 mm apical to the soft tissue margin with a needle for anaesthesia and a silicon disk stop with a 3‐mm diameter. The distance was determined with a caliper.	NR	1.75 (1‐2)^a^ 1.73 ± 0.62	3 (2‐3)^a^ 2.90 ± 0.74	0 (0.5‐2)^a^ −1.18 ± 0.98
Roccuzzo et al.	NR	NR	NR	NR	NR	NR	NR	NR
Tavelli et al. (a)	CAF: 1.18 ± 0.40 TUN: 1.42 ± 0.42	CAF: 2.65 ± 0.50 TUN: 2.44 ± 0.34	CAF: 1.48 ± 0.47 TUN: 1.02 ± 0.49	The measurement was taken 1.5 mm apical to the soft tissue margin using a needle for anaesthesia and a silicon disk stop. The distance was recorded with a digital caliper.	CAF: 1.09 ± 0.44^b^ TUN: 0.56 ± 0.25^b^	CAF: 1.96 ± 1.35 TUN: 1.79 ± 0.99	CAF: 4.54 ± 1.05 TUN: 3.43 ± 1.22	CAF: 2.57 ± 0.90 TUN: 1.57 ± 1.05
Tavelli et al. (b)	0.93 ± 0.12	2.51 ± 0.53	1.58 ± 0.61	Measured 1.5 mm apical to the soft tissue margin with a needle for anaesthesia and a silicon disk stop. The distance was determined using a digital caliper.	*Midfacial* 0 mm: 3.02 ± 1.97 1 mm: 3.15 ± 2.04 2 mm: 2.36 ± 1.62 3 mm: 2.19 ± 1.21 4 mm: 2.47 ± 0.97 5 mm: 2.11 ± 1.05 6 mm: 1.48 ± 0.96 7 mm: 1.57 ± 0.85 8 mm: 1.59 ± 0.90	2.40 ± 0.77	3.55 ± 0.60	1.15 ± 1.06

References	Probing depth (mm)			Clinical attachment level (mm)			Attached mucosa width (mm)^c^			Bleeding on probing (%)		
	Baseline mean ± SD	Final mean ± SD	Baseline‐Final mean ± SD	Baseline mean ± SD	Final mean ± SD	Baseline‐Final mean ± SD	Baseline mean ± SD	Final mean ± SD	Baseline‐Final mean ± SD	Baseline mean ± SD	Final mean ± SD	Baseline‐Final mean ± SD
Anderson et al.	NR	NR	NR	NR	NR	NR	NR	NR	NR	NR	NR	NR
Zucchelli et al.	2 (2–2.9)^a^ 2.28 ± 0.70	2 (2‐2)^a^ 2.03 ± 0.57	0.21 ± 0.75	15.75 (14.62‐17)^a^ 16 ± 1.65	13 (12–14.50)^a^ 13.08 ± 1.53	3 (2.5–3.5)^a^ 2.97 ± 1.01	NR	NR	NR	NR	0	NR
Roccuzzo et al.	2.7 ± 0.4	2.9 ± 0.5	−0.2 ± 0.46	NR	NR	NR	NR	NR	NR	15.38	16.67	NR
Tavelli et al. (a)	CAF: 2.14 ± 0.41 TUN: 2.04 ± 0.46	CAF: 2.18 ± 0.54 TUN: 2.25 ± 0.51	CAF: 0.04 ± 0.50 TUN: 0.14 ± 0.50	CAF: 4.61 ± 1.06 TUN: 4.39 ± 0.81	CAF: 2.43 ± 0.76 TUN:3.18 ± 0.93	CAF: 2.18 ± 1.05 TUN: 1.21 ± 0.87	CAF: 0.43 ± 0.81 TUN: 0.29 ± 0.38	CAF: 2.36 ± 1.25 TUN: 1.29 ± 0.83	CAF: 1.93 ± 0.92 TUN: 1.00 ± 0.71	NR	NR	NR
Tavelli et al. (b)	2.35 ± 0.47	2.17 ± 0.41	0.17 ± 0.75	4.95 ± 0.69	2.58 ± 0.49	2.17 ± 0.68	0.40 ± 0.52	1.33 ± 0.68	0.75 ± 0.94	NR	NR	NR

*Note*: Publications with the longest follow‐up were selected.^12^, ^21^

Abbreviation: ADM, acellular dermal matrix allograft; CAF, coronally advanced flap technique; CTG, connective tissue graft; NR, not reported; PSTD, peri‐implant soft tissue dehiscence; SD, standard deviation; TUN, tunnel technique.

^a^ Data reported as median (quartiles).

^b^ Thickness of reconstructed volume. Volumetric changes were evaluated using digital impressions captured with intraoral optical scanning at baseline and 1 year after. The region of interest was defined as a rectangular area, with the soft tissue margin as its coronal border and extending 7 mm in the corono‐apical direction. The volumetric outcomes were expressed as the mean distance between the surface/mean thickness of the reconstructed volume in mm.

^c^ Calculated with the following formula: KMW–PD.

#### 3.3.1 Primary outcomes

##### Esthetic outcomes

Professionally assessment of esthetics was conducted in all the included studies at baseline^19^, ^22^, ^23^ and at different times throughout the investigation period [6 weeks,^23^ 3 months,^23^ 6 months,^23^, ^24^ 1 year,^19^, ^22^, ^24^, ^25^ 5 years,^12^, ^20^ and 10 years^21^]. Various methodologies were used to present the results, including the Complex Esthetic Index (CEI),^23^, ^26^ the Pink and White Esthetic Score (PES/WES),^12^, ^19^, ^29^ the Visual Analogue Scale (VAS, 0–10),^20^, ^21^, ^22^ and the Implant Soft Tissue Dehiscence Coverage Esthetic Score (IDES).^24^, ^25^, ^30^

**FIGURE 2:**
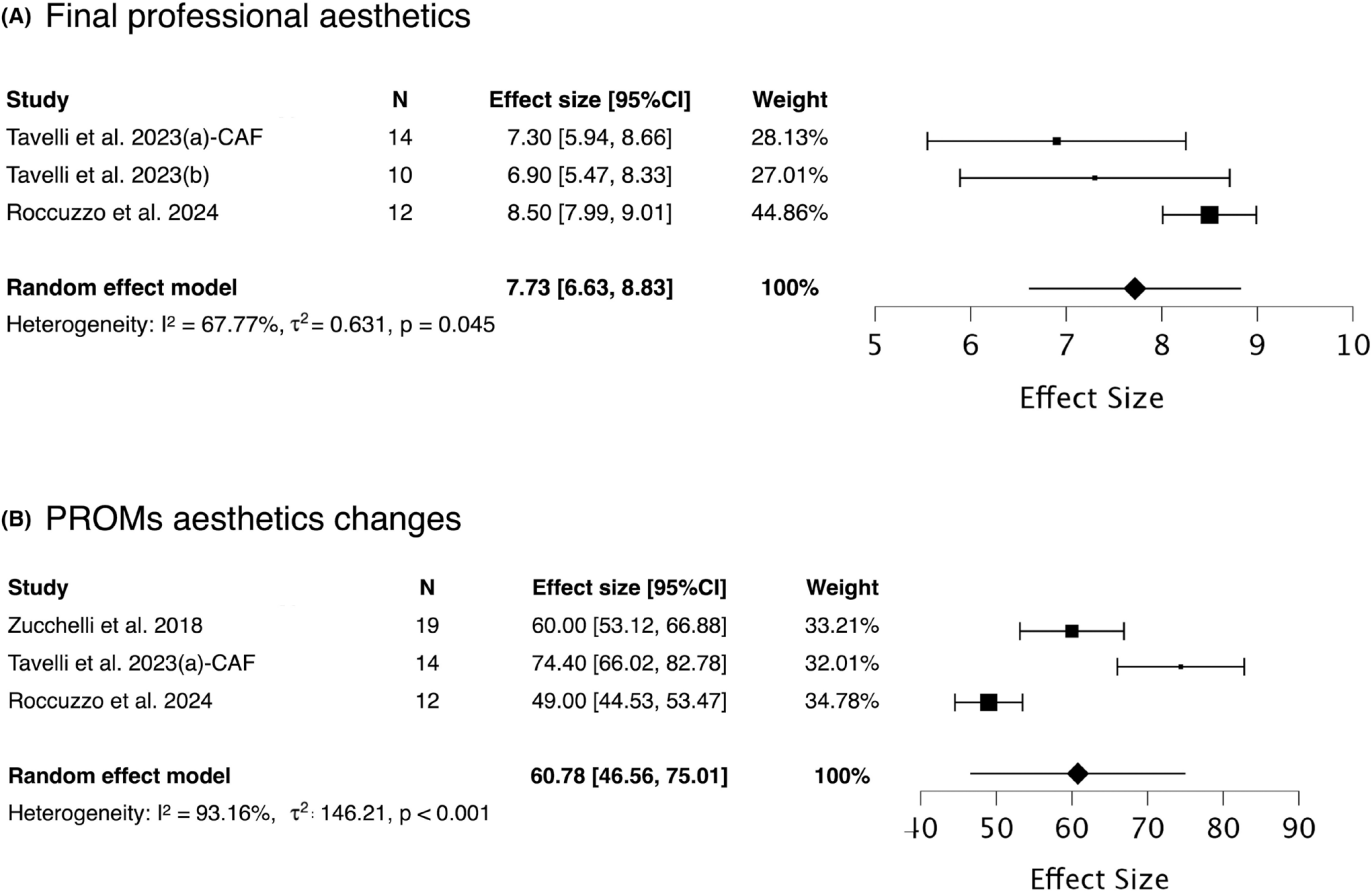
Meta‐analyses evaluating the effect of soft tissue augmentation procedures using a coronally advanced flap for peri‐implant soft tissue dehiscence in terms of both final professional esthetic scores and patient‐reported esthetic changes.

With respect to professional evaluation of esthetics, only three studies^21^, ^24^, ^25^ were included in the meta‐analysis, since different scales were reported. Moreover, when data from the same study were published more than once, the publication that provided the longest follow‐up^21^ was selected. The estimated mean value for professional‐reported esthetics outcomes was 7.7 (*n* = 3); 95% CI: [6.6; 8.8] based on a 0–10 scale, with moderate heterogeneity across the studies (*I* ^2^ = 67.77%; *T* ^2^ = 0.63) (Figure 2A). Regarding patient perception of esthetics, three studies^12^, ^21^, ^24^ were included in the meta‐analysis, which measured changes up to the end of treatment. Again, the publications with the longest follow‐up were selected. The estimated change in patient‐perceived esthetics was 60.8 (*n* = 3; 95% CI: [46.6; 75.0]), with a high heterogeneity across the studies (*I* ^2^ = 93.16%; *T* ^2^ = 146.21) (Figure 2B).

##### Patient satisfaction

Additionally, patient perception of esthetics was assessed in all included studies using a Visual Analogue Scale [VAS 0–10^12^, ^19^, ^20^, ^21^, ^25^; VAS 0–100^24^], except for Anderson et al.,^23^ who utilized the patient‐modified CEI.^26^ In this sense, the esthetic evaluation by patients was performed at the same time points as the professional assessment, with two exceptions: Tavelli et al.^24^ in which esthetics was evaluated at baseline and after 1 year of follow‐up, and Roccuzzo et al.,^20^, ^21^ where it was assessed only at 5 and 10 years after the surgical procedure.

Patient satisfaction was reported in two studies using either a VAS 0–100 (i.e., anxiety regarding the appearance of the dental implant)^24^ or a revised version of the Kiyak Post‐Surgical Patient Satisfaction Questionnaire.^23^, ^31^ However, due to the different methodologies employed, a meta‐analysis could not be conducted. In the study by Anderson et al.,^23^ specific data on patient satisfaction with treatment outcomes were not provided; nevertheless, the authors indicated that patients treated with CAF + CTG experienced a decrease in quality of life over a 6‐month period, whereas those treated with CAF + ADM showed an improvement in quality of life; however, differences between groups were not statistically significant. Additionally, Tavelli et al.^24^ observed a significant reduction in anxiety following PSTD treatment, with a greater improvement when the CAF technique was used compared to the TUN technique.

#### 3.3.2 Secondary outcomes

##### Patient‐related outcomes

Regarding patient morbidity, three studies^23^, ^24^, ^25^ evaluated postoperative pain during the first two weeks after the surgical procedure. Pain was measured using a VAS^24^, ^25^ or a questionnaire with a 5‐point scale.^23^ However, due to variations in methodology, a meta‐analysis could not be performed.

##### Changes in soft tissue thickness

**FIGURE 3:**
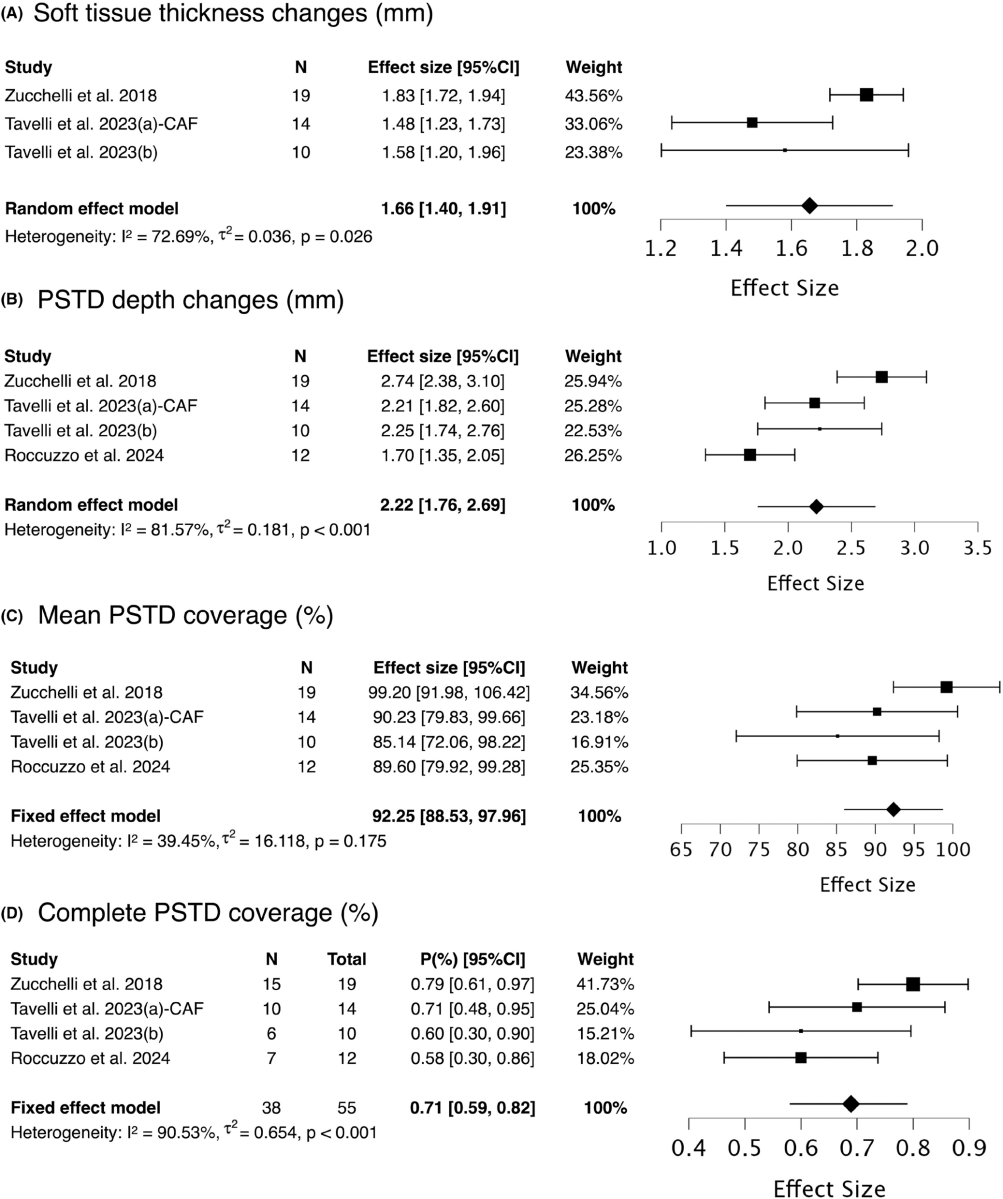
Meta‐analyses assessing the effect of soft tissue augmentation procedures using a coronally advanced flap for peri‐implant soft tissue dehiscence (PSTD) on soft tissue thickness changes, PSTD depth changes, mean PSTD coverage, and complete PSTD coverage.

Soft tissue thickness (STT) changes were evaluated in four studies.^12^, ^23^, ^24^, ^25^ Anderson et al.^23^ determined STT using transmucosal probing with an endodontic file and a stopper at 1 and 3 mm apical to the soft tissue margin, while the other three studies used a short needle for anesthesia and measurements were recorded 1.5 mm apical to the mucosal margin.^12^, ^24^, ^25^ Due to differences in methodology and missing data (i.e., one study did not report SD), only three studies^12^, ^24^, ^25^ were included in the meta‐analysis. The estimated gain of STT was 1.7 mm (*n* = 3; 95% CI: [1.4; 1.9]) (*I* ^2^ = 72.69%; *T* ^2^ = 0.04) (Figure 3A).

##### Changes in the position of peri‐implant soft tissue margin

Changes in the PSTD depth were evaluated in all studies by measuring the distance from the peri‐implant soft tissue margin to the cemento‐enamel junction of the homologous contralateral tooth^12^, ^21^, ^24^, ^25^ or to the crown margin^23^ using a periodontal probe. However, due to missing information in one investigation,^23^ only four studies were included in the meta‐analysis. The estimated reduction of PSTD depth was 2.2 mm (*n* = 4; 95% CI: [1.8; 2.7]) (*I* ^2^ = 81.57%; *T* ^2^ = 0.18) (Figure 3B).

Data on mean PSTD coverage were derived from four studies that reported outcomes at 1 year,^24^, ^25^ 5 years,^12^ and 10 years^21^ of follow‐up. The findings indicated an estimated PSTD coverage of 92.3% (*n* = 4; 95% CI: [88.5; 97.9]) (*I* ^2^ = 39.45%; *T* ^2^ = 16.12). Moreover, the estimated rate of complete PSTD coverage was 71% (*n* = 4; 95% CI: [59; 82]) (*I* ^2^ = 90.53%; *T* ^2^ = 0.65) (Figure 3C, D).

##### Changes in the keratinized mucosa

Keratinized mucosa (KM) changes were assessed in three studies.^12^, ^24^, ^25^ The measurements were taken from the mucosal margin to the mucogingival junction at the mid‐buccal aspect using a periodontal probe^12^, ^24^, ^25^ and a resin stent.^12^, ^24^ The meta‐analysis estimated a KM gain of 1.6 mm after the surgical intervention (*n* = 3; 95% CI: [0.7; 2.6]) (*I* ^2^ = 90.53%; *T* ^2^ = 0.65) (Figure S1A).

##### Clinical parameters

Changes in probing depth and clinical attachment level were recorded in four^12^, ^21^, ^24^, ^25^ and three^12^, ^24^, ^25^ studies, respectively. While probing depth remained stable (*n* = 4; 0.0 mm; 95% CI: [−0.1; 0.2]) (*I* ^2^ = 30.04%; *T* ^2^ = 0.01), an estimated clinical attachment gain of 2.5 mm was observed (*n* = 3; 95% CI: [1.9; 2.9]) (*I* ^2^ = 73.88%; *T* ^2^ = 0.16) (Figure S1B,C).

Finally, the attached mucosa was obtained in two studies by calculating the difference between KM width and PD using a periodontal probe^24^, ^25^ and customized stents.^24^ The estimated gain of attached mucosa was 1.4 mm (*n* = 2; 95% CI: [0.2; 2.5]) (*I* ^2^ = 89.31%; *T* ^2^ = 0.62) (Figure S1D).

### 3.4 Quality assessment of the included studies

The results of quality assessment are presented in Figures S2 and S3.

The risk of bias for one of the RCTs was rated as low,^24^ while the other RCT study was assessed as having a high risk of bias,^23^ primarily due to some concerns regarding the randomization process, missing outcome data, outcome measurements, and the selection of the reported results.

Among the observational cohort studies, two were considered as good,^12^, ^19^, ^20^, ^21^, ^22^ while the remaining study was rated as fair.^25^

Inter‐examiner reliability was almost perfect for RCT assessment (k score = 0.90, 95% CI: 0.81–0.99) and for prospective case series assessment (k score = 0.82, 95% CI: 0.48–1.00).

## 4 DISCUSSION

The primary aim of this study was to systematically evaluate the performance of soft tissue augmentation procedures at dental implants affected by buccal PSTD, focusing on professional evaluation of esthetics as well as self‐reported patient esthetics perceptions and satisfaction.

Soft tissue augmentation procedures around implants with buccal PSTD demonstrated a positive impact on esthetics based on professional evaluations, achieving an average score of 7.7 on a 0–10 scale, with moderate heterogeneity across studies (*I* ^2^ = 67.77%). This heterogeneity may be attributed to differences in esthetic scales, surgical techniques, and prosthetic modifications. Although several validated scales and indices are available to evaluate implant esthetics—including the Papilla Index,^32^ the Implant Crown Esthetic Index (ICAI),^33^ the Pink and White Esthetic Score (PES/WES),^29^ the Complex Esthetic Index (CEI),^26^ the Implant Soft Tissue Dehiscence Coverage Esthetic Score (IDES),^30^ and the Visual Analogue Scale (VAS, 0–10)—there is no consensus on the most reproducible esthetic score. To standardize procedures, the meta‐analysis included only studies using the CAF technique with a 10‐point scale (IDES and VAS) for professional esthetic assessments.

Notably, the study by Roccuzzo et al.^21^ reported an esthetic score over one point higher than those observed in the studies by Tavelli et al..^24^, ^25^ This inferior esthetic score reported in the latter studies might be explained by the use of a more objective and sophisticated IDES scale compared to VAS. In this context, IDES, specifically developed for the professional evaluation of PSTD therapy, assesses aspects such as soft tissue margin level, peri‐implant papillae height, as well as peri‐implant mucosa color and appearance.^30^ Another possible reason for the lower esthetic scores observed in the study by Tavelli et al.^25^ may be the initial interproximal attachment loss on adjacent teeth. This condition is a common esthetic concern for patients and a critical prognostic factor that limits vertical reconstruction of soft tissues.^34^ Additionally, the use of vertical releasing incisions in the CAF surgical technique^24^, ^25^ as compared to the modified CAF^22^ could also contribute to these lower esthetic outcomes. However, Tavelli et al.^24^ found that the CAF technique achieved significantly higher IDES scores compared to the TUN technique, primarily due to improved scores in the level of the mucosal margin and peri‐implant mucosal appearance. Apart from a proper management of soft tissues, the esthetic success of a PSTD coverage is closely linked to the implant restoration. Since prosthetic modifications were only performed in two of the included studies,^12^, ^25^ the potential scientific benefit of a surgical‐prosthetic approach from an esthetic standpoint could not be thoroughly evaluated through pair‐wise meta‐analyses. Removal of the prosthetic component can provide enhanced surgical access, facilitating precise graft placement and potentially leading to superior soft tissue adaptation and more predictable esthetic outcomes.^18^ Additionally, the absence of the restoration minimizes mechanical interference, allowing for optimal tissue healing. However, temporary removal may negatively impact patient comfort and satisfaction during the healing period, particularly in esthetically sensitive areas. Furthermore, the need for prosthetic disconnection and reconnection can introduce additional costs and complexity to the treatment process.

Likewise, patients' esthetic perception experienced a moderate improvement after soft tissue augmentation of PSTD, achieving an average change of 60.8 on a 100‐mm VAS, although high heterogeneity across studies was observed (*I* ^2^ = 93.16%). While all studies included in the meta‐analysis evaluated patient esthetics using the VAS, this substantial variability may be attributed to differences in individual esthetic expectations before and after treatment. Although buccal PSTD has a minimal impact on self‐perceived esthetics,^2^ factors such as initial PSTD height and the magnitude of coverage achieved, particularly in anterior sites, likely influence patient expectations after PSTD therapy. Thus, it is not surprising that Roccuzzo et al.^21^ reported the smallest change in patient esthetic satisfaction, given their study's relatively shallow initial PSTD depth (1.9 ± 0.7 mm) and lower rate of complete dehiscence coverage (58.3%). The choice of the donor site for soft tissue grafting may have also played a role in influencing clinical and esthetic outcomes. Harvesting grafts from the maxillary tuberosity provides a denser, more fibrous connective tissue, which can increase the potential for long‐term volume preservation.^35^ However, this tissue type has been associated with a tendency for a more hyperplastic healing response, which may compromise the esthetic outcome by leading to excessive tissue thickness or irregular contours.^36^, ^37^

Furthermore, the patient‐reported esthetic satisfaction following PSTD therapy was not consistent with the professional esthetic evaluation, as patients rated the esthetic outcomes more favorably than professionals. This discrepancy, also observed in the evaluation of implant‐supported restorations in the anterior maxilla^38^, ^39^, ^40^ and in root coverage procedures,^41^, ^42^ may be attributed not only to the professionals' more objective and technical perspective compared to patients' subjective judgments, but also to the lack of standardized tools for measuring professional and patient implant esthetics.^40^ It can be also mentioned that for professional esthetics evaluation, data from the study of Zucchelli et al.^12^ were not included in the meta‐analysis due to the use of PES/WES score.

As mentioned before, various approaches (i.e., surgical and restorative) have been proposed to address PSTD. However, evidence remains limited on the most effective and predictable technique in terms of reduction of PSTD depth and, subsequently, increasing patient esthetic satisfaction. Among the surgical techniques described are as follows: (i) coronally advanced flap, with or without vertical releasing incisions, combined with CTG or soft tissue substitutes (STS), (ii) envelope flap, pouch, or tunnel techniques with CTG or STS, (iii) submerged techniques, with or without CTG; (iv) free gingival grafts, and (v) guided bone regeneration.^9^ Additionally, a modified laterally positioned flap technique has been investigated as a potential approach for the treatment of PSTD.^43^

In this study, the estimated reduction of PSTD depth was 2.2 mm (95% CI: [1.76; 2.69]). It is noteworthy that only groups treated with a CAF (with or without vertical releasing incisions) were included in the analysis to minimize potential bias. The most commonly documented technique in the literature for achieving complete coverage of buccal gingival recessions involves combining a CAF with CTG.^44^, ^45^ Indeed, while TUN is effective for treating both localized and multiple gingival recessions, CAF appears to be associated with a higher percentage of complete root coverage when compared to the TUN technique.^46^ Therefore, it seems reasonable to extrapolate these findings to the treatment of PSTD. In this context, Tavelli et al.^24^ suggested considering several critical surgical factors: (a) flap elevation from the implant surface and adjacent bone/periosteum can present challenges with the TUN technique, (b) achieving adequate flap release and removing muscle fibers as well as residual tension are often challenging in the presence of bulky crowns that restrict the angulation of tunneling blades into the sulcus, and (c) CAF facilitates split‐thickness flap elevation, allowing the periosteum, anatomical papillae, and connective tissue fibers attached to the implant to serve as anchorage for the CTG. In contrast, in sites treated with the TUN technique, the CTG is secured only to the flap which may lead to apical displacement if flap healing is suboptimal. It is worth noting that recent designs of the TUN technique include VISTA or distant incisions in the mucosal tissue, which facilitate the coronal advancement of the flap.

Overall, findings from the present meta‐analysis, based on four studies, indicated an estimated PSTD coverage of 92.2% and a complete PSTD coverage rate of 71%. These results are in agreement with those obtained in a recent systematic review, which reported a complete coverage rate of 70%.^10^ In contrast, Burkhardt et al.,^47^ in a prospective cohort study, reported no cases of complete coverage among the ten PSTD cases treated. However, several factors may have influenced these outcomes. Notably, the authors did not specify the type of dehiscence or detail the presence of attachment loss in adjacent teeth—both critical factors that could impact treatment success. Furthermore, only three of the treated PSTDs had a depth of ≤2.5 mm, a parameter known to significantly favor complete coverage.^46^ Differences in graft harvesting techniques may also explain the divergent results. Burkhardt et al. utilized a subepithelial CTG, which tends to contain more fatty and glandular tissue, making it more susceptible to contraction compared to a deepithelialized free gingival graft.^46^, ^48^

Regarding changes in STT, the current meta‐analysis estimated a gain of 1.7 mm. In this context, it has been demonstrated that thin soft tissues (<2 mm) have been associated with significantly greater recession (i.e., 0.62 mm) compared to patients with a thick phenotype (≥2 mm).^49^ Notably, substantial evidence underscores the crucial role that increased STT plays in maintaining the long‐term stability of the soft tissue margin in natural teeth following root coverage procedures,^50^ suggesting that this principle may similarly apply to implant sites.^24^ Interestingly, Amid et al.^51^ found a significant correlation between STT and the reduction of dehiscence depth, highlighting the importance of assessing this factor prior to surgical intervention. It is important to note that, in the present meta‐analysis, there was a moderate heterogeneity in the results across the studies, which might be partially attributed to variations in methodologies and measurement techniques employed to assess STT. Moreover, some studies did not report graft thickness, which could be a critical factor influencing the gain in STT.

Interestingly, the presence of at least 1.5 mm of keratinized tissue following root coverage procedures has also been associated with the long‐term stability of the gingival margin.^50^ However, the impact of soft tissue grafting procedures for treating PSTD on the gain of KM remains poorly investigated, with limited evidence available in the literature. In the present study, the meta‐analysis estimated a clinically relevant gain of KM following surgical intervention (1.6 mm; 95% CI: [0.68; 2.61]). One possible explanation could be related to the tissue quality characteristics and the specific soft tissue harvesting site. In this regard, Bertl et al.^52^ demonstrated that variations in harvesting techniques yield grafts with different tissue compositions, specifically in the relative proportions of fibrous connective tissue (CT) and fatty glandular tissue (FGT). These findings may clarify previous observations regarding the potential of CTGs to induce epithelial keratinization. Some studies indicated that CTGs obtained from attached gingiva, mainly composed of fibrous CT, or from the superficial palatal layers, rich in lamina propria, tend to develop a keratinized epithelium post‐transplantation.^53^ Conversely, it has been demonstrated that sites receiving the deep CTG predominantly displayed a higher proportion of FGT.^54^ Notably, it should be considered that all included studies in this systematic review obtained CTGs through extraoral de‐epithelization, which may have influenced the observed outcomes.

Several limitations should be considered for a more accurate interpretation of the results. Firstly, the scarce number of studies evaluating the primary outcome and the small patient samples within studies are relevant factors. Added to this, moderate‐to‐high heterogeneity across studies was also observed, although the *I* ^2^ should be viewed cautiously due to the inclusion of very few studies. This heterogeneity was particularly notable due to variations in study populations, designs (i.e., RCTs and case series), inclusion criteria, and settings (i.e., university versus private practice), as well as differences in follow‐up durations, types and severities of PSTD, surgical‐prosthetic approaches (e.g., removal of the prosthetic restoration), soft tissue graft harvesting sites, and the time points and methods used for outcome assessments. Moreover, there are limited data on peri‐implant health over time and no available data on alternative therapies (e.g., implant removal or alveolar ridge reconstruction). Importantly, the results of the meta‐analysis are largely applicable to a single surgical technique—namely, the coronally advanced flap (CAF)—as nearly all studies or study arms included employed this approach, thereby limiting the generalizability of the findings to other surgical techniques. Finally, other patient‐related outcomes such as morbidity, quality of life, and treatment satisfaction after PSTD therapy were seldom reported.

It should also be noted that, due to surgical and anatomical challenges, and the absence of standardized techniques, adverse outcomes—such as failure to achieve buccal PSTD coverage or even deterioration relative to the baseline condition—may occur.

Given these considerations, upcoming research in this field should be focused on conducting:

- Studies that utilize a standardized, universally accepted, and validated method for professionals' esthetic assessment after PSTD therapy, alongside specific patient‐reported measures for subjective esthetic evaluation.
- More studies that uniformly evaluate other patient‐related outcomes, including post‐surgical morbidity, quality of life, and satisfaction (i.e., overall treatment time, costs) as well as other clinical variables such as peri‐implant soft tissue color changes and volumetric changes of peri‐implant soft tissue.
- More RCT studies that evaluate the efficacy of PSTD therapy in terms of patient‐related outcomes, comparing different surgical techniques, CTG harvesting sites, soft tissue substitutes, and prosthetic modifications.

## 5 CONCLUSIONS

Based on limited evidence, it can be concluded that CTG procedures around implants affected by buccal PSTD appear to positively influence both professional and patient‐reported esthetics outcomes. Further studies employing standardized tools for esthetic evaluation and patient satisfaction are needed to validate these findings.

## CONFLICT OF INTEREST STATEMENT

The authors declare that they have no conflict of interest. This Workshop is Organized by the European Federation of Periodontology, along with the Italian and Spanish Societies of Periodontology.

## Supporting information


Figures S1–S3.

## Data Availability

Data available on request from the authors.

## APPENDIX 1 SEARCH STRATEGY FOR PIOS QUESTION

In systemically healthy humans with at least one dental implant with a peri‐implant buccal soft tissue dehiscence (population), what is the performance of soft tissue augmentation procedures (intervention), in terms of professionally determined esthetic scores and self‐reported patient satisfaction (primary outcome), as reported in randomized controlled clinical trials, controlled clinical trials, prospective―cohort studies or case series―with at least 6 months of follow‐up (study design)?

### MEDLINE (via PubMed) search strategy

1. Dental Implants [mh].
2. Dental Implantation [mh].
3. Dental Prostheses, Implant‐Supported [mh].
4. Dental Implants, Single‐Tooth [mh].
5. Dental implant [title/abstract].
6. Dental implants [title/abstract].
7. Dental implantation [title/abstract].
8. Oral implant [title/abstract].
9. Oral implants [title/abstract].
10. Oral implantology [title/abstract].
11. Implant placement [title/abstract].
12. Soft tissue defect [title/abstract].
13. Soft tissue defects [title/abstract].
14. Soft tissue dehiscence [title/abstract].
15. Soft tissue dehiscences [title/abstract].
16. Soft tissue recession [title/abstract].
17. Soft tissue recessions [title/abstract].
18. Mucosal recession [title/abstract].
19. Mucosal recessions [title/abstract].
20. Esthetic complication [title/abstract].
21. Esthetic complications [title/abstract].
22. (#1 OR #2 OR #3 OR #4 OR #5 OR #6 OR #7 OR #8 OR #9 OR #10 OR #11 OR #12 OR #13 OR #14 OR #15 OR #16 OR #17 OR #18 OR #19 OR #20 OR #21)
23. Connective tissue [mh].
24. Connective tissue [title/abstract].
25. Subepithelial connective tissue [title/abstract].
26. Connective tissue graft [title/abstract].
27. Free gingival graft [title/abstract].
28. Gingival autograft [title/abstract].
29. Autograft* [title/abstract].
30. Autogenous graft [title/abstract].
31. Allograft [mh].
32. Allograft [title/abstract].
33. Acellular dermal matrix [title/abstract].
34. Dermal matrix allograft [title/abstract].
35. AlloDerm [title/abstract].
36. Acellular dermis [title/abstract].
37. Collagen matrix [title/abstract].
38. Collagen matrix xenograft [title/abstract].
39. Xenogeneic collagen matrix [title/abstract].
40. Collagen‐based matrix [title/abstract].
41. Soft tissue augmentation [title/abstract].
42. Soft tissue graft [title/abstract].
43. Soft tissue coverage [title/abstract].
44. Surgical coverage [title/abstract].
45. Recession coverage [title/abstract].
46. (#23 OR #24 OR #25 OR #26 OR #27 OR #28 OR #29 OR #30 OR #31 OR #32 OR #33 OR #34 OR #35 OR #36 OR #37 OR #38 OR #39 OR #40 OR #41 OR #42 OR #43 OR #44 OR #45)
47. Clinical trials, randomized [mh].
48. Controlled clinical trials, randomized [mh].
49. Controlled clinical trial [mh].
50. Clinical trial [mh].
51. Randomized controlled trial [pt].
52. Controlled clinical trial [pt].
53. Clinical trial [pt].
54. Random*
55. Trial
56. Cohort
57. Prospective
58. Case series
59. (#47 OR #48 OR #49 OR #50 OR #52 OR #53 OR #54 OR #55 OR #56 OR #57 OR #58)
60. (#22 AND #46 AND #59)

### Cochrane Central Register of Controlled Clinical Trials (CENTRAL) search strategy

1. MeSH descriptor: [Dental Implants] explode all trees.
2. MeSH descriptor: [Dental Implantation] explode all trees.
3. MeSH descriptor: [Dental Prosthesis, Implant‐Supported] explode all trees.
4. MeSH descriptor: [Dental Implants, Single‐Tooth] explode all trees.
5. “Dental implant*”:ti,ab,kw OR “Oral implant*”:ti,ab,kw OR “Implant placement”:ti,ab,kw.
6. “Soft tissue def*”:ti,ab,kw OR “Soft tissue dehis*”:ti,ab,kw.
7. {OR #1‐#6}.
8. MeSH descriptor: [Connective Tissue] explode all trees.
9. “Subepithelial connective tissue”:ti,ab,kw OR “Connective tissue graft”:ti,ab,kw.
10. MeSH descriptor: [Allografts] explode all trees.
11. “Acellular dermal matrix”:ti,ab,kw OR “Dermal matrix allograft”:ti,ab,kw OR “Alloderm”:ti,ab,kw OR “Acellular dermis”:ti,ab,kw OR Allograft:ti,ab,kw OR “Collagen matrix”:ti,ab,kw OR “Collagen matrix xenograft”:ti,ab,kw OR “Xenogeneic collagen matrix”:ti,ab,kw OR “Collagen‐based matrix”:ti,ab,kw OR “Soft tissue graft”:ti,ab,kw OR “Soft tissue coverage”:ti,ab,kw OR “Recession coverage”:ti,ab,kw {OR #8‐#9}.
12. {OR #8‐#11}.
13. (#7 AND #12)
14. (#13 NOT Pubmed)

### SCOPUS search strategy

1. KEY (“Dental implant*”) OR KEY (“Implant‐supported dental prosthesis”) OR KEY (“Single‐tooth implant*”).
2. TITLE‐ABS‐KEY (“Dental implant*”) OR TITLE‐ABS‐KEY (“Oral implant*”) OR TITLE‐ABS‐KEY (“Implant placement”).
3. TITLE‐ABS‐KEY (Soft W/2 def*) OR TITLE‐ABS‐KEY (Soft W/2 dehisc*) OR TITLE‐ABS‐KEY (“Soft tissue” W/2 rec*).
4. (#1 OR #2 OR #3)
5. KEY (“Connective tissue graft”).
6. KEY (“Allograft”).
7. TITLE‐ABS‐KEY (“Connective tissue”) OR TITLE‐ABS‐KEY (“Subepithelial connective tissue”) OR TITLE‐ABS‐KEY (Connective W/2 graft*) OR TITLE‐ABS‐KEY (“Gingival autograft*”) OR TITLE‐ABS‐KEY (Autograft*) OR TITLE‐ABS‐KEY (Soft tissue PRE/2 augmentation) OR TITLE‐ABS‐KEY (“Soft tissue graft*”) OR TITLE‐ABS‐KEY (“Allograft”) OR TITLE‐ABS‐KEY (“Acellular dermal matrix”) OR TITLE‐ABS‐KEY (“Dermal matrix allograft”) OR TITLE‐ABS‐KEY (“Alloderm”) OR TITLE‐ABS‐KEY (“Acellular dermis”) OR TITLE‐ABS‐KEY (“Collagen matrix”) OR TITLE‐ABS‐KEY (“Collagen matrix xenograft”) OR TITLE‐ABS‐KEY (“Collagen‐based matrix”).
8. (#5 OR #6 OR#7)
9. KEY (Random* W/2 trial) OR KEY (Control* W/2 trial) OR KEY (“Clinical trial”).
10. TITLE‐ABS‐KEY (Random* W/2 trial) OR TITLE‐ABS‐KEY (Control* W/2 trial) OR TITLE‐ABS‐KEY (“Clinical trial”) OR TITLE (Random*) OR TITLE (Trial*) OR TITLE (Prospective) OR TITLE (Cohort) OR TITLE (Case series).
11. (#9 OR #10).
12. (#4 AND #8 AND #11).

### EMBASE search strategy

(KEY (“Dental implant*”) OR KEY (“Implant‐supported dental prosthesis”) OR KEY (“Single‐tooth implant*”)) OR (TITLE‐ABS‐KEY (“Dental implant*”) OR TITLE‐ABS‐KEY (“Oral implant*”) OR TITLE‐ABS‐KEY (“Implant placement”)) OR (TITLE‐ABS‐KEY (soft W/2 def*) OR TITLE‐ABS‐KEY (soft W/2 dehisc*) OR TITLE‐ABS‐KEY (“Soft tissue” W/2 rec*)) AND (KEY (“Connective tissue graft”) OR KEY (“Allograft”) OR TITLE‐ABS‐KEY (“Connective tissue”) OR TITLE‐ABS‐KEY (“Subepithelial connective tissue”) OR TITLE‐ABS‐KEY (connective W/2 graft*) OR TITLE‐ABS‐KEY (“Gingival autograft*”) OR TITLE‐ABS‐KEY (autograft*) OR TITLE‐ABS‐KEY (soft AND tissue PRE/2 augmentation) OR TITLE‐ABS‐KEY (“Soft tissue graft*”) OR TITLE‐ABS‐KEY (“Allograft”) OR TITLE‐ABS‐KEY (“Acellular dermal matrix”) OR TITLE‐ABS‐KEY (“Dermal matrix allograft”) OR TITLE‐ABS‐KEY (“Alloderm”) OR TITLE‐ABS‐KEY (“Acellular dermis”) OR TITLE‐ABS‐KEY (“Collagen matrix”) OR TITLE‐ABS‐KEY (“Collagen matrix xenograft”) OR TITLE‐ABS‐KEY (“Collagen‐based matrix”)) AND (KEY (random* W/2 trial) OR KEY (control* W/2 trial) OR KEY (“Clinical trial”) OR TITLE‐ABS‐KEY (random* W/2 trial) OR TITLE‐ABS‐KEY (control* W/2 trial) OR TITLE‐ABS‐KEY (“Clinical trial”) OR TITLE (random*) OR TITLE (trial*) OR TITLE (prospective) OR TITLE (cohort) OR TITLE (case AND series)).

## APPENDIX 2 STUDIES EXCLUDED AFTER FULL‐TEXT READING, WITH REASON FOR EXCLUSION

**Table:**

Reference	Reason For Exclusion
Burkhardt et al. (2008)	1
Clem et al. (2023)	3
Farooqui et al. (2023)	2
Frisch and Ratka‐Krüger (2020)	1
Huang et al. (2021)	3
Lorenzo et al. (2012)	3
Qiu et al. (2023)	2
Rojo et al. (2020)	2
Stefanini et al. (2020)	1

Reason for exclusion (***Legend***):

1. No esthetic outcomes or PROMs.

2. STA performed prior to implant function.

3. Not aimed at PSTD improvement.

Abbreviations: PROM, patient‐reported outcome measure; PSTD, peri‐implant soft tissue dehiscence; STA, soft tissue augmentation.
