# Supplementary material for: Impact of soft tissue augmentation procedures on esthetics and patient satisfaction in the treatment of peri‐implant buccal soft tissue dehiscences: A systematic review and meta‐analysis
Source: Periodontol 2000. 2025 Jul 17;99(1):42–60. doi: 10.1111/prd.12633 (PMC13428092; doi:10.1111/prd.12633)
Supplement: Supplementary file 1 — Figures S1–S3. [file PRD-99-42-s001.zip › Figure Legends.docx]

**Figure S1** Meta-analyses examining the effect of soft tissue augmentation procedures using a coronally advanced flap for peri-implant soft tissue dehiscence (PSTD) on keratinized tissue (KM) width, probing depth (PD), clinical attachment level (CAL), and attached mucosa changes.

**Figure S2** Risk of bias assessment for randomized clinical trials.

**Figure S3** Risk of bias assessment for prospective case series.
