# Supplementary figures and images for: Impact of soft tissue augmentation procedures on esthetics and patient satisfaction in the treatment of peri‐implant buccal soft tissue dehiscences: A systematic review and meta‐analysis
Source: Periodontol 2000. 2025 Jul 17;99(1):42–60. doi: 10.1111/prd.12633 (PMC13428092; doi:10.1111/prd.12633)

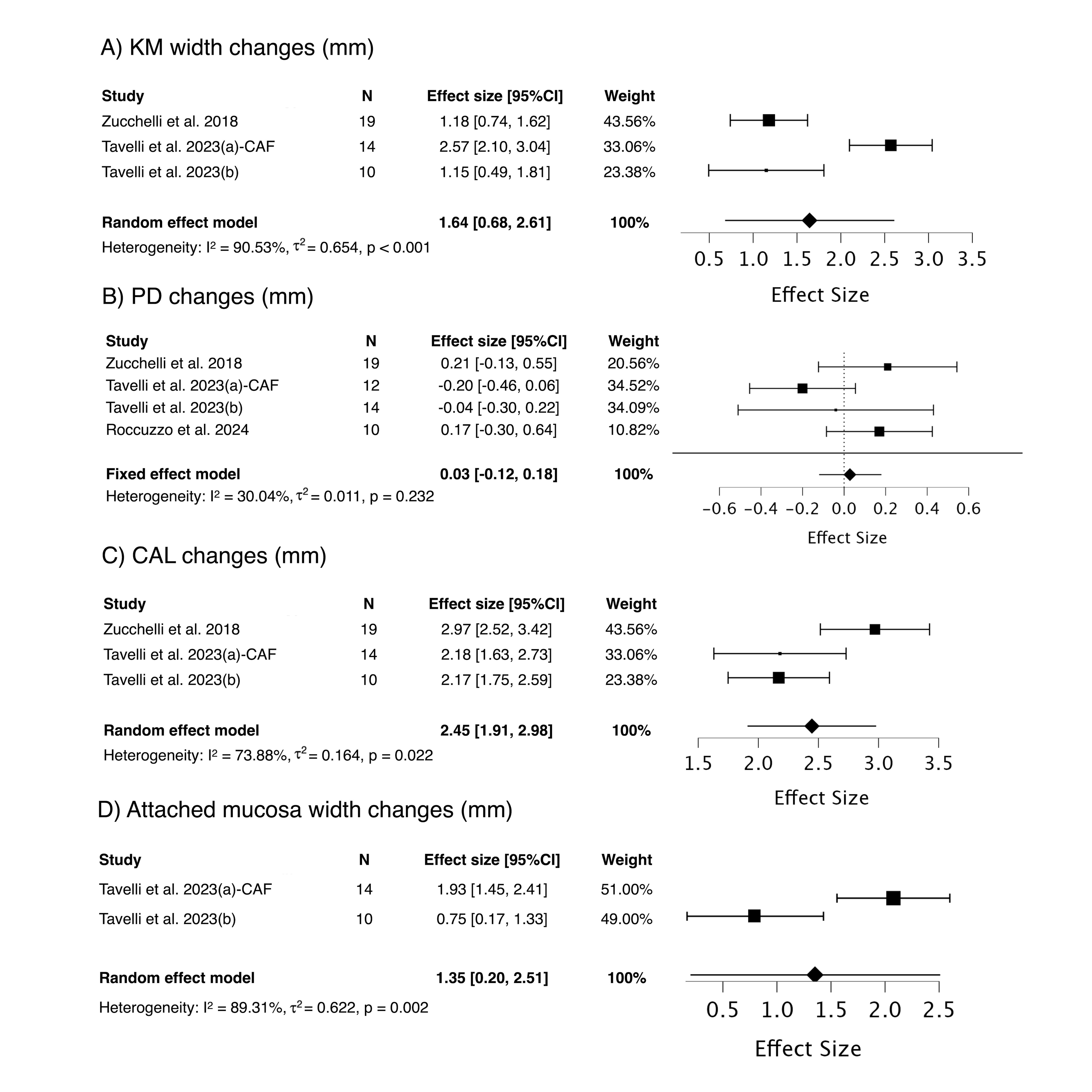

Supplement: Supplementary file 1 — Figures S1–S3. [file PRD-99-42-s001.zip › prd12633-sup-0001-Figs1.jpeg]

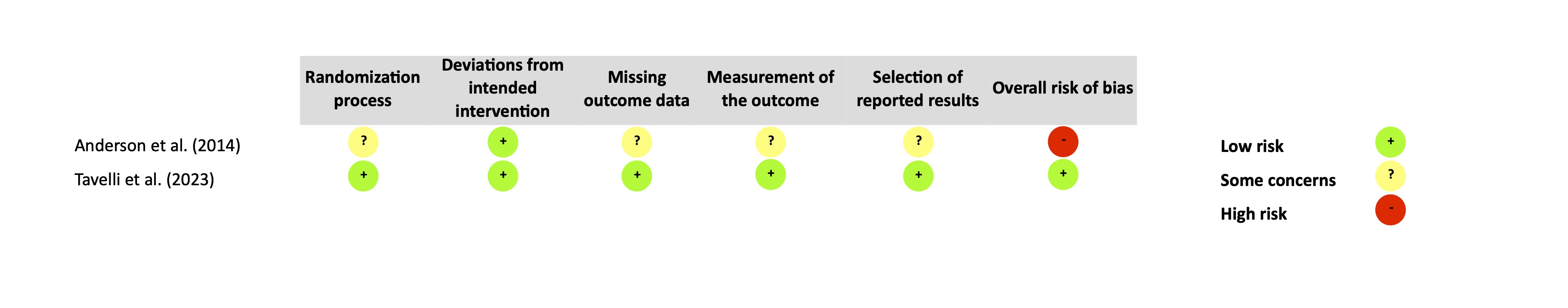

Supplement: Supplementary file 1 — Figures S1–S3. [file PRD-99-42-s001.zip › prd12633-sup-0002-Figs2.tiff]

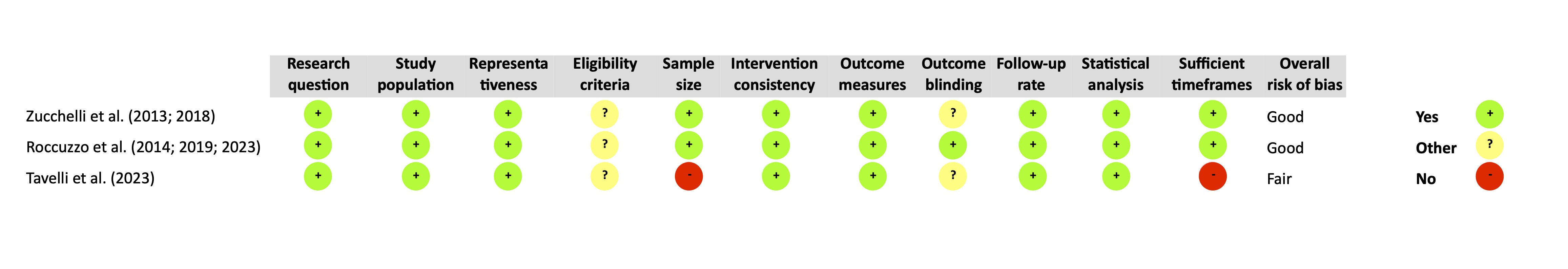

Supplement: Supplementary file 1 — Figures S1–S3. [file PRD-99-42-s001.zip › prd12633-sup-0003-Figs3.tiff]
